# Current Understanding of Molecular Pathology and Treatment of Cardiomyopathy in Duchenne Muscular Dystrophy

**DOI:** 10.3390/molecules20058823

**Published:** 2015-05-15

**Authors:** Tirsa L. E. van Westering, Corinne A. Betts, Matthew J. A. Wood

**Affiliations:** Department of Physiology, Anatomy and Genetics, University of Oxford, South Parks Road, Oxford, OX1 3QX, UK; E-Mails: tirsa.vanwestering@dpag.ox.ac.uk (T.L.E.W.); corinne.betts@dpag.ox.ac.uk (C.A.B.)

**Keywords:** heart, dystrophin, calcium, nNOS, mitochondria, utrophin up-regulation, read-through, viral gene therapy, cell-based therapy, exon skipping

## Abstract

Duchenne muscular dystrophy (DMD) is a genetic muscle disorder caused by mutations in the *Dmd* gene resulting in the loss of the protein dystrophin. Patients do not only experience skeletal muscle degeneration, but also develop severe cardiomyopathy by their second decade, one of the main causes of death. The absence of dystrophin in the heart renders cardiomyocytes more sensitive to stretch-induced damage. Moreover, it pathologically alters intracellular calcium (Ca^2+^) concentration, neuronal nitric oxide synthase (nNOS) localization and mitochondrial function and leads to inflammation and necrosis, all contributing to the development of cardiomyopathy. Current therapies only treat symptoms and therefore the need for targeting the genetic defect is immense. Several preclinical therapies are undergoing development, including utrophin up-regulation, stop codon read-through therapy, viral gene therapy, cell-based therapy and exon skipping. Some of these therapies are undergoing clinical trials, but these have predominantly focused on skeletal muscle correction. However, improving skeletal muscle function without addressing cardiac aspects of the disease may aggravate cardiomyopathy and therefore it is essential that preclinical and clinical focus include improving heart function. This review consolidates what is known regarding molecular pathology of the DMD heart, specifically focusing on intracellular Ca^2+^, nNOS and mitochondrial dysregulation. It briefly discusses the current treatment options and then elaborates on the preclinical therapeutic approaches currently under development to restore dystrophin thereby improving pathology, with a focus on the heart.

## 1. Introduction

Duchenne muscular dystrophy is a severe muscle wasting X-linked genetic disease which affects 1 in 3500 children [1]. It is characterized by loss in muscle function leading to decreased ambulation and most patients succumb to cardiac or respiratory failure [2,3]. The disease is caused by the almost complete absence of the dystrophin protein due to out-of-frame mutations in the *DMD* gene [4]. Dystrophin is an important protein for cytoskeletal structure and normal muscle function and plays a vital role in membrane stability and signaling [5].

Patients with DMD suffer from severe cardiomyopathy. Although the degree of cardiac involvement varies between patients, cardiomyopathy generally manifests at about 10 years of age and is prevalent in most patients by 20 years of age [6]. These patients first exhibit left ventricle (LV) dilation and hypertrophy, which progresses to a stage known as dilated cardiomyopathy (DCM). Additional cardiomyopathic features include decreased fractional shortening and electrocardiogram (ECG) abnormalities [7,8,9,10]. Approximately 26% of patients also display tachycardia, and 51% exhibit an increase in pathological heart rate variability [7]. These ECG abnormalities are associated with morphological changes to cardiac muscle (thin and thick filaments of heart) [11], myocardial fibrosis and conduction defects [12]. Indeed, deep Q wave abnormalities have been associated with lateral wall scarring [13].

The cardiac phenotype continues to deteriorate as dystrophic hearts undergo hypertrophy in an effort to cope with the increase in wall stress imposed by pressure/volume overload. The heart further undergoes maladaptive remodeling, developing DCM [6]. Approximately 25% of patients under the age of 6 present with DCM, which escalates to 59% by 10 years of age and is prevalent in all patients by adulthood [8]. Unfortunately, severe scoliosis hampers the ability to accurately measure cardiac function which results in late diagnosis and late employment of treatment [6,14,15].

Marked decline in cardiac function correlates with increased fibrotic deposition, impeding normal heart contraction. Fibrotic involvement is prevalent from a young age (17% of patients under 10 years), and escalates with age (34% between 10–15 years and 59% older than 15 years of age) [16].

The most widely used mouse model of DMD is the *mdx* mouse which has a point mutation in exon 23, thus preventing the production of dystrophin protein [17,18]. This model exhibits an obvious skeletal phenotype including elevated creatine kinase (CK) plasma levels, irregular muscle histology [17], muscle necrosis [19], and a decline in specific force and power with age [20]. Additionally, respiratory function deteriorates and correlates with degeneration of the diaphragm as the disease progresses with age.

*Mdx* mice also display a cardiac phenotype. Increased right ventricle (RV) systolic volume and subsequent reduction in RV ejection fraction (EF) is seen at 3 months [21]. An increase in myocardial fibrosis is apparent from 6 months of age, whilst LV cardiac output (CO) deteriorates. By 9 months of age, stroke volume (SV) is reduced followed by a reduction in LV EF at 12 months. Although this model has been criticized for the late onset cardiac phenotype, it should be noted that, at 1 month of age, *mdx* mice exhibit early intolerance to dobutamine stress [21]. As with DMD patients, ECG recordings in conscious *mdx* mice revealed tachycardia, decreased heart rate variability [22], deep Q waves, diminished S:R ratios, polyphasic R-waves and shorter QT and PR intervals [23] compared to controls.

The major disparity between *mdx* mice and DMD patients is that RV involvement does not occur in all DMD patients and cardiac complications contribute significantly to early mortality in patients. Interestingly BMD patients also exhibit RV changes prior to LV, analogous to *mdx* cardiac progression. DMD patients generally receive early ventilatory intervention due to the rapid deterioration in respiratory function. This intervention would be likely to improve pulmonary hypertension and thus may compensate for RV dysfunction.

It is thus evident that the heart is an important system affected in DMD. More recently, research has focused on characterizing heart pathology and therefore this review focuses on the cardiac aspects of DMD. First the result of the absence of dystrophin on molecular heart pathology will be discussed, particularly concentrating on the role of intracellular calcium (Ca^2+^) increase, the perturbed nitric oxide (NO) signaling and neuronal NO synthase (nNOS) function and the role of mitochondria within DMD. It should be noted that many other cellular pathways are important in DMD pathology (e.g., inflammation, matrix metalloproteinase function, autophagy and apoptosis), but will not be discussed here (see e.g., Shin *et al.* [24] and Mosquiera *et al.* [25]). Additionally, current therapies involving corticosteroids, respiratory support, ACE-inhibitors, beta-blockers and cardiac resynchronization techniques are reviewed. Lastly, preclinical therapeutic approaches that are presently investigated, such as exon skipping and viral gene therapy, will be explored within the context of improving heart pathology.

## 2. Molecular Pathology of DMD

**Figure 1:**
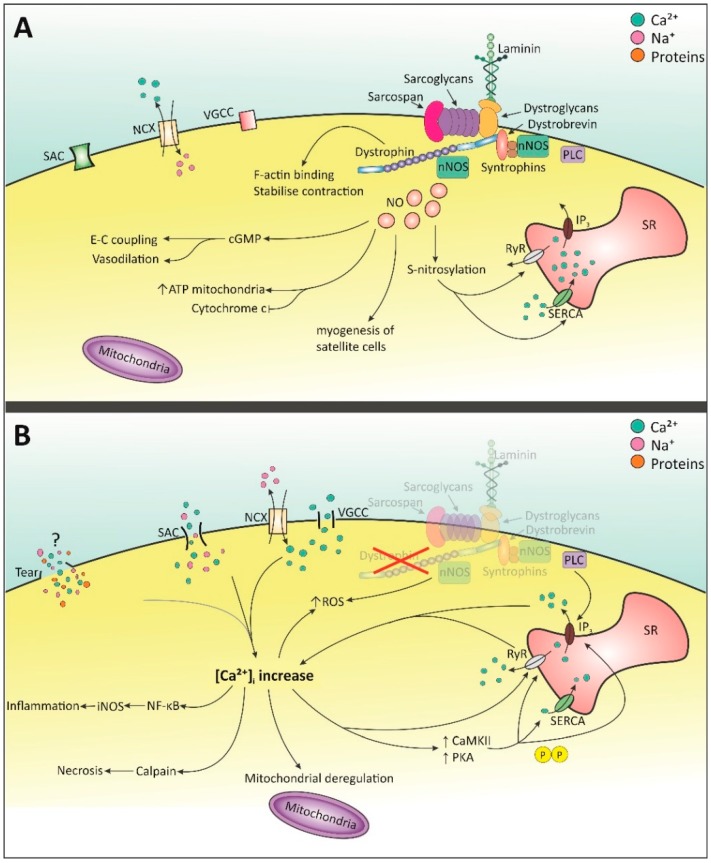
Molecular pathways involving Ca^2+^ and NO signaling in healthy and dystrophic cell. (**A**) Dystrophin present: Ca^2+^ is balanced by SACs, NCX and VGCC. nNOS is activated by Ca^2+^-dependent calmodulin [40,41,42]. NO is produced which is involved in many processes including normal E-C coupling, and S-nitrosylation of SERCA and RyR [25,40,42,43]. Upon E-C coupling, [Ca^2+^]_i_ rises via opening of RyR and IP_3_ receptor. When the stimulus stops, intracellular Ca^2+^ returns to normal via SERCA and other cell membrane Ca^2+^-permeable channels. (**B**) Dystrophin absent: membrane tears, SACs, NCX and VGCC increase intracellular Ca^2+^ [28,35,36,38,44,45]. Elevated Ca^2+^ increases CaMKII and PKA which hyper-phosphorylate Ca^2+^ channels on the SR [31,32,33]. SERCA levels may decrease and RyR becomes sensitive to Ca^2+^ [31,32,46,47,48]. Mis-localization of nNOS disrupts NO signaling and function [49,50]. This combined with raised Ca^2+^ increases ROS production [28,51]. Increased [Ca^2+^]_i_ elicits multiple detrimental events [24,30]. SACs: stretched-activated channels; NCX: Na^+^-Ca^2+^ exchanger; VGCC: Voltage-gated Ca^2+^ channels; nNOS: neuronal nitric oxide synthase; SERCA: Sarcoplasmic/endoplasmic reticulum Ca^2+^-ATPase; RyR: ryanodine receptor; NO: nitric oxide; IP_3_: inositol 1,4,5-triphosphate; SR: sarcoplasmic reticulum; CaMKII: calmodulin-kinase II; PKA: protein kinase A.

It is clear that dystrophin plays an important role in the cell. By connecting with laminin at the C-terminus through the dystroglycan complex, actin at the N-terminus, and spectrin-like repeat 11–17 in the rod domain, dystrophin provides stability to the cell and prevents damage from muscle contraction [5,26,27]. It is also involved in signaling through its association to the dystroglycan complex (DGC) at the cysteine-rich domain and C-terminus and neuronal nitric oxide synthase (nNOS) at its rod domain (see Figure 1) [5]. The location of mutation/deletion within the dystrophin gene correlates with the severity of cardiomyopathy. Deletions which affect the amino-terminal domain (muscle promoter, exon 1 or intronic regions) are associated with early-onset DCM, whereas deletions in the rod domain and hinge 3 region result in later onset DCM (mid-40’s) [8]. Absence of this protein, as is the case in DMD, renders both skeletal and cardiac cells more susceptible to damage upon muscle contraction. Increased permeability of the cell membrane has also been observed, possibly due to lipid peroxidation by phospholipase A_2_ or reactive oxygen species (ROS). This allows larger proteins, such as CK, to traverse the cell membrane [28]. Furthermore, many signaling pathways within the cell are affected and these factors lead to an imbalance in the intracellular environment, further resulting in cell muscle damage and eventually necrosis. The ensuing muscle pathology is characterized by cell degeneration and regeneration, whereby muscle cells are eventually replaced by fibrotic tissue [19].

### 2.1. Intracellular Ca^2+^ Increase 

One of the first events thought to influence molecular pathology is the increase in intracellular Ca^2+^. Given the importance of Ca^2+^ in excitation-contraction (E-C) coupling and as a signaling molecule, this has a detrimental effect on heart function and Ca^2+^ downstream pathways. The heart exhibits a smaller Ca^2+^ influx than skeletal muscle, but this smaller influx has a greater influence on cardiac cells, suggesting perhaps a more sensitive Ca^2+^-induced Ca^2+^ release [29]. Elevated intracellular Ca^2+^ in the heart leads to mitochondrial deregulation, protease calpain-mediated necrosis and NF-κB activation, which induces transcription of inducible NOS (iNOS)-mediated inflammation [24,30]. In addition, it activates Ca^2+^/calmodulin (CaM) and CaM kinase II (CaMKII) and protein kinase A (PKA) which hyper-phosphorylate Ca^2+^ channels [31,32,33]. Seval calcium binding proteins are known to be affected in the skeletal muscle phenotype. However, only two have been discussed in heart pathology in DMD, namely calsequestrin and sarcalumenin, which are involved in sequestration and SERCA Ca^2+^ uptake, respectively, and are both decreased in *mdx* hearts [34].

As of yet it is unclear how the increase in Ca^2+^ commences, particularly the initial entry of Ca^2+^. Many studies have focused on this topic and membrane tears and stretch-activated channels (SACs) have been proposed as possible mechanisms. More indirect entry of Ca^2+^ from the extracellular space is thought to be contributed by the Na^+^-Ca^2+^-exchanger (NCX) and voltage-gated Ca^2+^ channels (VGCC). The initial intracellular Ca^2+^ increase brings about the activation of store-operated channels (SOCs) such as sarcoplasmic/endoplasmic reticulum Ca^2+^ ATPase (SERCA), the ryanodine receptor (RyR) and the inositol 1,4,5-triphosphate (IP_3_) receptor (IP_3_R), thereby further increasing the intracellular Ca^2+^ concentration ([Ca^2+^]_i_) (see Figure 1).

#### 2.1.1. Membrane Tears

Membrane tears were suggested to arise from a fragile membrane due to the absence of dystrophin [35]. This would make the membrane leaky, allowing the influx and efflux of ions and proteins [28,36] (see Figure 1). Within the heart, a membrane sealant lowered, but not abolished, increased Ca^2+^ influx and improved left ventricular end diastolic volume in *mdx* mice [37,38]. More recently, the contribution of membrane tears to increased Ca^2+^ influx has been debated. In an extensive review, Allen and Whitehead indicated that the time required for Ca^2+^ to increase (10–20 min after contraction) did not correlate with membrane tears [39]. The leakage of ions and proteins through the membrane can also be explained by SACs and by lipid peroxidation [28]. Given these opposing results, more research is needed to elucidate these findings.

#### 2.1.2. Stretched-Activated Channels (SACs)

More recently, SACs have been investigated as a possible reason for the initial Ca^2+^ influx [36]. These channels open upon stretched muscle contraction and allow the influx of cationic ions [52]. Inhibition of SACs following exposure to mechanical stress significantly diminished the rise of [Ca^2+^]_i_ in ventricular cardiomyocytes and restored protein kinase C (PKC) and phospholipase C (PLC) signaling [53,54]. It should be noted that the non-specificity of the blockers used means they may have also been blocking Na^+^ channels [53]. Therefore, the increase in [Ca^2+^]_i_ could be mediated by both direct entry of Ca^2+^ ions through SACs, and indirect entry through the Na^+^/Ca^2+^-exchanger (NCX) or voltage-gated Ca^2+^ channels (VGCC) that open due to Na^+^-induced membrane depolarization (see Figure 1) [38,53,55,56,57]. SACs are composed of multiple channels and subunits, some of which have been implicated in the composition of these SACs and DMD pathology. These channels include the mechano-sensitive transient receptor potential cation (TRPC) channels, namely TRPC1, TRPC3, TRPC6 and TRPV2.

TRPC1 channels are activated by the ROS-dependent Src kinase leading to an influx of Ca^2+^. Both the absence of dystrophin-associated nNOS [51] as well as the increase in [Ca^2+^]_i_ [28] can induce increased ROS levels. The role of TRPC1 in the heart remains elusive, but the expression levels of this channel increase with age in the *mdx* mouse [53]. Interestingly, a more recent experiment did not see an involvement of TRPC1 channels in skeletal myotubes [54]. Whether this is also the case in cardiomyocytes is unclear. TRPV2 channels have also been implicated in DMD and upon activation migrate to the plasma membrane. Cultured *mdx* cardiomyocytes exhibit increased TRPV2 expression at the plasma membrane, and inhibition of these channels with non-specific blockers and targeted siRNAs reduced cellular Ca^2+^ influx upon osmotic stress [52]. TRPC6 channels are also sensitive to stretch contraction [58] and inhibition of this channel in the *mdx* heart reduced contractions and arrhythmias [59]. Finally, relevance of TRPC3 in the heart remains to be established as TRPC3 knock-out (KO) mice exhibit a dystrophic phenotype, but stretch-induced contraction of TRPC3-/- cardiomyocytes does not induce a Ca^2+^ response [59,60].

#### 2.1.3. Voltage-Gated Ca^2+^ Channels (VGCC)—l-Type

The voltage-dependent l-type Ca^2+^ channel has been implicated in the pathology of DMD. This Ca^2+^ channel is linked to F-actin by subsarcolemmal proteins including dystrophin and α-actinin [61,62] and is thought to be involved in contraction [63]. In *mdx* mouse hearts, the l-type Ca^2+^ channel exhibits a delayed inactivation rate and interestingly, even though action potential duration is not affected, significant ECG abnormalities are observed [64]. Additionally, Viola *et al.* showed that communication between l-type Ca^2+^ channels and mitochondria was important for metabolic function in healthy cells and this communication was altered in *mdx* cardiomyocytes, though the exact proteins involved are unclear [44,45].

#### 2.1.4. Store-Operated Ca^2+^ Release

Other intracellular channels on the sarcoplasmic reticulum (SR) involved in regulating [Ca^2+^]_i_ are SERCA, RyR and IP_3_ channels (see Figure 1). Being a major store of Ca^2+^, SERCA pumps Ca^2+^ back into the SR when [Ca^2+^]_i_ increases [65]. Interestingly, expression levels of SERCA in the *mdx* heart appear to be similar to that of wild-type (WT), though other reports have shown down-regulation [46,47]. This may be attributed to age, where older mice would exhibit a reduction in SERCA channels. RyRs are activated by Ca^2+^ binding, thereby leading to Ca^2+^-induced Ca^2+^ release stimulating excitation-contraction (E-C) coupling [65,66]. Hyper-phosphorylation of the channel, possibly by protein kinase A (PKA) or CaMKII, could make it more sensitive to Ca^2+^ [31,48,66]. Persistent hyper-phosphorylation of RyR leads to dissociation from calstabin 2, a stabilizing protein [67]. This in turn disrupts Ca^2+^ regulation whereby the SR becomes leaky, ultimately resulting in heart failure [33]. Indirect evidence of RyR involvement in the *mdx* heart is seen, whereby increased SR Ca^2+^ leak was observed in 3–4 and 12–15 month old mice, with a worsened pathology in the older mice [68]. These events are also observed in arrhythmias and heart failure [69]. Lastly, IP_3_ receptors are activated by IP_3_, a downstream product of PLC. Application of PLC inhibitors reduced the Ca^2+^ concentration in the heart of *mdx* mice compared to WT. Older *mdx* animals (9 and 12 months) showed a larger diastolic Ca^2+^ decrease upon inhibition compared to WT. These results indicate the involvement of PLC and its downstream effects on IP_3_ and diastolic Ca^2+^ concentration in the heart [69]. Moreover, it also indicates that age plays an important role in the increases of intracellular Ca^2+^ concentration.

Much work has been done to elucidate the role of Ca^2+^ in DMD pathology, which suggests the interaction of multiple mechanisms. It is possible other uncovered avenues play an important role in addition to the mechanisms proposed so far.

### 2.2. NO and NOS

An important second messenger in cell physiology is the signaling molecule nitric oxide (NO). Its production is regulated by NO synthase (NOS) of which there are three isoforms: neuronal NOS (nNOS), endothelial NOS (eNOS) and inducible NOS (iNOS). nNOS and eNOS are both constitutively expressed in muscle and heart and activated by the Ca^2+^-dependent calmodulin. eNOS is associated with caveolin 3 on the sarcolemma and nNOS is associated with the DGC and is in close proximity to SERCA and RyR [40,41,42,70]. Conversely, iNOS is only expressed in the cell during inflammation and partly responsible for the inflammatory response seen in DMD [40,41]. The localization of NOS enzymes within the cell is crucial to their function as NO is only active for several seconds and thus needs to reach its target quickly [42]. However, in DMD nNOS is displaced, with considerably lower nNOS activity levels in patients and *mdx* mice [49,50].

The function of nNOS in the heart is currently still being investigated. Several knock-out studies have implicated a role in LV systolic function and shortening of myocytes, but this remains elusive due to opposing results [42,71,72]. Overproduction of NO by nNOS is associated with cardiac depression, which increases the chances of heart failure [49]. Absence of nNOS impacts several downstream pathways, including the mitochondrial respiratory chain, RyR regulation and reactive oxygen species (ROS) control, which induces cell damage and further increases Ca^2+^ [25,40,41,72,73]. Transgenic up-regulation of nNOS in the *mdx* mouse restored heart phenotype, with a decrease in inflammatory markers, fibrosis and apoptosis. Some aspects of heart function also improved, such as stroke volume and ejection fraction [74]. Together these studies underscore the importance of nNOS and NO function and their interaction with [Ca^2+^]_i_ in the heart and loss of these molecules results in worsening of heart function, though further research is needed to elucidate its exact function.

### 2.3. Mitochondrial Dysfunction

In any cell, mitochondria are an important part of energy metabolism and healthy muscle contraction [65]. In addition to this role, it also functions as a Ca^2+^ store, supplying and taking up Ca^2+^ to and from the cell [75,76]. Furthermore, it is involved in ROS production and plays an important role in cell death through necrosis and apoptosis [77]. *Mdx* mice display disturbed mitochondrial function and thus these organelles are thought to play a role in DMD.

Altered mitochondrial energy production is one of the first pathophysiological changes known to occur in *mdx* heart. *Mdx* mice were assessed at 10–12 weeks of age, considered the pre-cardiomyopathic stage, and revealed a slight shift in energy consumption, from the normally utilized fatty acids to a higher usage of carbohydrates [78]. The functional oxidative capacity of the mitochondria is also disturbed in the *mdx* mouse model. It is suggested that mild oxidative stress induces a reduction in oxidative phosphorylation, thereby reducing the production of ATP. Due to the energy demand of the cell and the inability of the mitochondria to supply the cell, cardiomyocytes are severely affected [43]. Mitochondria are also a target of NO signaling and its downstream effectors. Overexpression of guanylyl cyclase (GC; activated by cGMP), in 12 and 20 week old *mdx* mice improved muscle function and cardiac endurance and led to reduced heart production of lactate dehydrogenase, a marker for tissue damage. The GC transgenic *mdx* mouse heart still displayed a slight shift in energy metabolism from fatty acids to carbohydrate substrates, but mitochondrial function was enhanced as higher levels of the compounds for the citric acid cycle were observed [79]. Given this data, the lack of dystrophin seems to disrupt metabolism in the heart. However, it remains unclear what brings about this change in metabolism and whether this is a compensatory or detrimental mechanism [78].

In addition to the perturbed energy metabolism exhibited by mitochondria in dystrophin-deficient cells, the increased intracellular Ca^2+^ levels have a detrimental effect on the mitochondria. The rising [Ca^2+^]_i_ is thought to induce a sudden increase in mitochondrial membrane permeability through the mitochondrial permeability transition pore (PTP). The PTP is a voltage-sensitive channel and an increase in the mitochondrial Ca^2+^ concentration induces the opening of this channel, leading to swelling. Short-term opening of these channels is thought to be beneficial as the mitochondria are able to regulate intracellular Ca^2+^ and ROS balance [80,81]. However, long-term opening of this channel is thought to be detrimental to the cell and induces necrosis of the mitochondria [82]. The ruptured mitochondria contributes to myofiber necrosis [56].

## 3. Current Clinical Disease Management and Application of Commercially Available Drugs

There is currently no cure for DMD, and therefore therapy is limited to the management of symptoms. The three predominant treatment approaches include corticosteroids in order to ameliorate skeletal muscle phenotype, mechanical respiratory support and cardiac assessment and treatment. Corticosteroids have been shown to markedly improve muscle strength and function [83]. More recent studies have implicated corticosteroids in the stabilization of pulmonary function, prolonging ambulation and reducing the prevalence of scoliosis [84]. In addition they have been associated with delaying the onset of cardiomyopathy by 4% for each year of corticosteroid treatment [85]. However, the unfortunate side effects including Cushing’s syndrome, short stature, hypertension, hyperglycemia, cataracts and osteoporosis, hampers the use of corticosteroids [86]. 

Deterioration in respiratory muscles results in respiratory insufficiency and pulmonary hypertension. An increase in pulmonary hypertension compounds cardiac function leading to DCM [87]. Thus mechanical support is required as soon as nocturnal hypoventilation is apparent, in order to maintain adequate ventilation and coughing reflex [88]. A non-invasive positive-pressure ventilation (NIPPV) machine with a mechanical insufflation-exsufflation device is the preferred respiratory support device and has substantially reduced mortality in DMD patients [89].

Lastly, the approaches designed to specifically target cardiac involvement include angiotensin-converting enzyme (ACE) inhibitors, beta-blockers (β-blockers) and diuretics [90]. ACE inhibitors are generally implemented when LV systolic function declines [3]. These inhibitors prevent the conversion of angiotensin-I to angiotensin-II (Ang-II), thereby reducing circulating levels of the Ang-II. In addition, ACE inhibitors reduce kinase-II and increase bradykinin levels, which have been shown to reduce peripheral vascular resistance, improve endothelial function and prevent fibrosis. The use of ACE inhibitors for the treatment of DMD patients with an ejection fraction (EF) below 55%, showed a significant improvement in cardiac function (relative to the pre-therapy results; *p* < 0.0001) and delayed the progression of cardiomyopathy [91]. 

Beta-Blockers prevent the binding of catecholamines by interfering with β-receptor binding, thus reducing sympathetic nervous system activity. They are therefore prescribed for arrhythmic patients, and those with symptomatic but stable systolic dysfunction. Clinicians often prescribe a cocktail of ACE inhibitors, β-blockers and diuretics which significantly improves cardiac function and survival in DMD patients [91,92]. However, regular cardiac assessments are imperative to ensure the correct treatment regimen, as it has been observed that administration of the β-blocker metoprolol was detrimental to RV function when administered at early stages of cardiomyopathic progression [93].

In DMD patients whom are unresponsive to pharmacological treatment, cardiac resynchronization therapy may be considered, *i.e.*, patients with low LV EF, accompanied by ventricular dyssynchrony (QRS > 120 ms) [94]. These patients have a bi-ventricular pacemaker surgically installed, which allows the pacing of the septal and lateral walls of the LV. Other measures such as left ventricular assist devices HeartMate II and HeartWare, have also been employed in an effort to reduce heart failure [95]. In both cases these devices markedly improve cardiac function and quality of life, thus demonstrating their potential for DMD patients who do not respond to conventional interventional measures.

Several drugs that are already commercially available may benefit DMD patients and have therefore been investigated in animal models of DMD to explore their therapeutic potential. The synthetic copolymer poloxamer P188, which stabilizes the sarcolemma, has been shown to improve the cardiac phenotype and even prevent isoproterenol/dobutamine-induced cardiomyopathy/death [37,96]. The phosphodiesterase 5 (PDE5) inhibitor, sildenafil, acts on the cGMP pathway, where it enhances NO-cGMP signaling. This signaling is impaired in *mdx* mice and administration of sildenafil improved contractile performance, cardiac metabolism and sarcolemmal integrity [97]. Furthermore, it reversed pathological cardiac dysfunction in *mdx* hearts [98], and improved diaphragmatic muscle strength thereby enhancing respiratory function [99]. Unfortunately, when translated to clinic, no cardiac function improvement was observed in patients and the clinical trial was terminated early [100,101]. It is important to note, however, that a relatively small cohort of patients was used in this study and higher numbers may better elucidate the underlying mechanisms. In addition, the drugs losartan and pirfenidone have been investigated for their anti-fibrotic properties. These drugs inhibit TGF-β expression, thereby reducing fibrosis and they were seen to improve cardiac pathology and function [102,103]. Pharmacological measures to target the NO signaling pathway have also been utilized and shown to slower the muscular dystrophy progression. Indeed, histone deacteylase activity (HDAC), which is perturbed in DMD may be inhibited with the use of NO donors, and has also been shown to benefit heart pathology [104]. In addition the combination of a non-steroidal anti-inflammatory drug, ibuprofen, and an NO donor, isosorbide dinitrate, were capable of preventing morphological changes in the heart of *mdx* mice [105]. A recent phase 1 clinical trial using this combinatory therapy, demonstrated an excellent safety profile in healthy patients [106]. This provides a solid basis for further trials in muscular dystrophy patients.

These drugs have demonstrated encouraging results in animal models of DMD and it is hoped that they will infer some clinical benefit in patients. Furthermore, these drugs may present viable combinatory therapies which may be administered in conjunction with treatments specifically aimed at correcting/compensating for the dystrophin mutation.

## 4. Preclinical Therapeutic Approaches

Given that current treatment strategies are limited to reducing the symptoms of the disease, it is vital that new therapies are developed that directly target the underlying mutation. Several very promising strategies currently under investigation for treating DMD also have potential for treating the heart. This review will discuss some of these most promising therapies, namely; utrophin up-regulation, ribosomal read-through therapy, stem cell therapy, viral gene therapy delivering mini/micro-dystrophin and exon skipping antisense oligonucleotides. Not all experiments regarding the different approaches are discussed in this review, but a summary of selected references are cited in Table 1.

**Table 1:** *DMD therapies under development.* Several strategies have been employed to further develop the different types of therapies (specific strategy), which are in different stages of research, either clinical or pre-clinical (research stage and selected models). A brief summary of the results of these strategies is mentioned (results of therapy) with selected references. *IV: Intravenous; IM: intramuscular; IP: intraperitoneal*.

Therapy	Specific Strategy	Research Stage and Selected Models	Results of Therapy	Selected References
Utrophin up-regulation	Utrophin transgene	Preclinical—mdx/utrn^−/−^	Transgenic utrophin expression which improved pathology in skeletal muscle, but not heart.	[107,108,109]
	Zinc fingers	Preclinical—cultured cells, mdx muscle	Successful activation of utrophin improved muscle function and reduced pathology in TA. No heart data.	[110,111,112]
	Biglycan	Preclinical—mdx	Localizes utrophin to sarcolemma. Treatment reduced pathology in quadriceps and diaphragm and improved physiology in EDL. No heart data.	[113]
	SMT C1100	Preclinical—mdx; Clinical trials—Phase Ia and Ib	Preclinically: increased RNA and protein of utrophin in skeletal and cardiac muscle. Reduced pathology and improved muscle function in skeletal muscle. Phase Ia: mild side-effects at higher dose. Phase Ib: no data.	[114,115]
Read-through therapy	Gentamicin	Preclinical—mdx; Clinical trials—Phase I	Preclinically: Low levels of dystrophin expression, including in heart, protection against muscle damage in EDL. Clinical trials: inconclusive.	[116,117]
	Negamycin	Preclinical—mdx	Antibiotic drug to reduce side effects seen in gentamycin. Subcutaneous injections negamycin safer than gentamycin, but induced low dystrophin expression in skeletal muscle and heart.	[118]
	PTC124	Preclinical—HEK293 cells and mdx; Clinical trials—Phase I, 2a/b	Preclinically: 20%–25% increase in dystrophin in TA, diaphragm and heart. Improved physiological function of EDL. Clinical trials: Generally well tolerated. Overall no significant improvement, but certain subgroups responded well to treatment.	[119,120,121,122]
	RTC13/RTC14	Preclinical—mdx	RTC13 demonstrated better efficacy (restored dystrophin in skeletal muscle and heart) than gentamicin, PTC124 and RTC14. Improved muscle function and decreased serum CK.	[123]
Viral gene therapy	Lentivirus	Preclinical—myotubes, primary myoblasts and mdx	Transfection with mini- or microdystrophin: 20%–25% dystrophin expression in TA muscles (for 2 year period). Less central nucleation, but no protection from muscle injury. Able to transfect TA myogenic progenitor cells.	[124,125,126]
	‘Gutted’ adenovirus	Preclinical—mdx	IM with full dystrophin cDNA displayed dystrophin expression, improved muscle force and protected against muscle damage.	[127]
	rAAV2/AAV8	Preclinical—mdx	Chimeric vector containing codon-optimized micro-dystrophin. IV injection resulted in almost 100% transfection, effective dystrophin expression in skeletal muscle and heart and improved muscle function. No immunological response was observed.	[128]
	rAAV6	Preclinical—mdx/utrn^−/−^ and mdx	Microdystrophin rAAV6 administered in 1 month old mdx/utrn^−/−^ increased life span, improved pathology and dystrophin (1 year post-injection). Dystrophin restored in heart and heart mass normal, but function not recovered. 20 mo mdx (4 months after injection) showed dystrophin expression in skeletal muscle and heart and improved pathology.	[129,130]
	AAV9	Preclinical—GRMD and mdx	IV mini-dystrophin administration to GRMD revealed varied dystrophin expression, also in heart. Micro-dystrophin administration in young mdx induced dystrophin expression and slowed progression of cardiac phenotype. 10 mo mice expressed dystrophin and cardiac function improved.	[131,132,133]
Cell-based therapy	Myoblasts	Preclinical—mdx Clinical trials	Ability to differentiate into myotubes. Preclinically: partial dystrophin expression in mdx mice. No heart data. Clinical trial: no beneficial effects	[134,135]
	Fibroblasts	Preclinical—mdx	Ability to differentiate into myotubes. Effective transfection with dystrophin expression in immunocompromised mice. No heart data.	[136]
	Bone marrow-derived stem cells	Preclinical—mdx and GRMD	Migrate to damaged muscle areas, differentiate into myogenic cells and aid regeneration. Substantial dystrophin restoration in skeletal muscle of mdx, but no restoration in GRMD dogs. No heart data.	[137,138]
	Cd133+ stem cells	Preclinical—scid/mdx; Clinical trial Phase I	Ability to differentiate into myocytes. Preclinically: effective dystrophin restoration in scid/mdx. No heart data. Clinical trial demonstrated safety.	[139,140]
	Mesangio-blasts	Preclinical—GRMD	Improved functional mobility and partial dystrophin restoration in skeletal muscle. No heart data.	[141]
	iPS cells	Preclinical—immuno-compromised mdx	Differentiating iPS cells into muscle precursor cells followed by injection into TA induced dystrophin expression. Cells integrated with muscle cells and settled in satellite cell population. Improved TA function. No heart data.	[142,143,144]
Antisense oligonucleo-tides	2′O MePS	Preclinical—mdx; Clinical trial Phase III	Preclinical: IM revealed low dystrophin restoration, even with multiple high doses. Clinical: 6 mg/kg was maximal tolerated dose in patients. Phase III trial did not meet 6MWD endpoint.	[145,146,147,148]
	PMO	Preclinical—mdx; Clinical trial Phase IIb	Preclinical: repeat IV administrations of high dose restored dystrophin in multiple skeletal muscles of the mdx mouse, <2% in heart. Clinically: well tolerated and dystrophin present after 48 weeks. At 84 weeks stabilization in the 6MWD; 120 weeks stabilized pulmonary function.	[149,150,151,152,153] (Sarepta press release, February 2014)
	Tricyclo-DNA	Preclinical- mdx	Multiple IV administrations and very high doses (200 mg/kg per week) resulted in dystrophin in skeletal muscle and heart, with low levels in the brain and improvements in cardiac and pulmonary function.	[154]
	Octa-guanidium conjugated PMO	Preclinical- mdx and GRMD	Capable of restoring dystrophin in skeletal muscle and hearts of mdx mice. This has further been demonstrated in dystrophic dogs. High doses led to adverse events in GRMD.	[155,156]
	CPP-AOs- Arginine rich	Preclinical—mdx and mdx/utrn^−/−^	(RXR)_4_ multiple IP produced ~100% dystrophin in diaphragm and low levels in skeletal muscles. Single IV restored dystrophin in skeletal muscle and diaphragm, ~50% in the heart. Improved mortality rate and corrected kyphosis in mdx/utrn^−/−^. (RXRRBR)_2_: Less toxic, repeat and high dose IV illustrated impressive exon skipping notably in heart (72%). Improvements in cardiac function, with preserved diastolic function after 6 months	[157,158,159,160,161,162,163,164]
	CPP-AOs- Pips	Preclinical—mdx	Pip2a and Pip2b: strong exon skipping following IM. Following IV, Pip5e induced high dystrophin restoration body wide including heart. Pip6-PMO series: Pip6a, Pip6b and Pip6f exhibited best dystrophin expression in heart. Long-term IV administration prevented deterioration in heart function in the event of exercise.	[165,166,167]
	CPP-AOs- Phage Peptides	Preclinical—mdx	MSP enhanced *in vivo* skeletal and cardiac muscle binding capacity. B-MSP-PMO showed 2–5 fold improvement in skeletal muscle compared to B-PMO (no dystrophin in heart). T-9 (SKTFNTHPQSTP) specificity in mdx quad and improved specificity over MSP. 12-mer phage resulted in ~25% dystrophin expression in skeletal muscle (75 mg/kg). A 7-mer phage conjugated to 2′OMePS resulted in exon skipping in multiple tissues including heart and diaphragm.	[168,169,170,171,172].

### 4.1. Utrophin Up-Regulation

Utrophin up-regulation was one of the first therapeutic strategies developed to compensate for the lack of dystrophin in the cell. In the adult cell, utrophin is mainly found in regenerating cells and at the neuromuscular and myotendinous junctions [173]. It is thought that utrophin could be important for determining actin filament length during development [174]. Utrophin is a homologue of dystrophin with a molecular weight of 395 kDa and a similar amino acid composition, although the rod domain is significantly shorter [175]. Despite functional differences, utrophin appears to have many of the same binding proteins, including certain components of the DAPC and F-actin [173,174,176]. Given these characteristics, it is believed that utrophin could partially compensate for dystrophin function and as such it has been considered a viable option for therapeutic purposes.

Consequently, up-regulation of utrophin to ameliorate DMD pathology and improve the functional deficit is being investigated. Evidence supporting this concept comes from the dystrophin/utrophin double knockout (*mdx*/utrn^-/−^) mouse model. These animals exhibit worsened skeletal muscle and cardiac pathology and have a life span of only 2–3 months [162,177,178,179], indicating that utrophin compensates for the absence of dystrophin in these animals. The positive effects of utrophin up-regulation are also seen in *mdx* skeletal muscle, though it should be noted that the heart is not corrected [107,108,109,110,111,112]. Another strategy called biglycan localizes utrophin to the sarcolemma, though no new utrophin is generated [113] (Tivorsan Pharmaceuticals: www.trivorsan.com).

The most promising utrophin targeting drug is SMT C1100, a drug discovered from a small molecule screen, currently undergoing clinical trials. Application of this drug in *mdx* mice increased utrophin RNA and protein levels, and the mice exhibited a reduced pathology and improved muscle function. The heart also displayed the presence of utrophin, but no functional tests were performed [114]. Phase Ia and Ib clinical trials have been performed, where the phase Ia trials showed that mild side-effects only occurred at higher doses [115]. The phase Ib trials have been completed, but results have yet to be published (clinicaltrials.gov, identifier: NCT02056808) (see Table 1).

It should be noted that utrophin compensation is unable to completely restore pathology. For instance, utrophin is unable to bind nNOS, a key factor in DMD pathophysiology [180]. Therefore, given the structural differences compared to dystrophin, it is not yet clear if utrophin can fully compensate for the absence of dystrophin. Nevertheless, utrophin up-regulation remains a very valuable therapeutic option as it could benefit all patients, irrespective of the underlying mutation causing the disease.

### 4.2. Stop Codon Read-Through Therapy

A small number of patients have a nonsense mutation which leads to a premature stop-codon (about 10%–15%) thereby disrupting the open reading frame [181,182]. Antibacterial drugs called aminoglycosides are able to ‘read-through’ these premature stop codons without affecting normal stop codons, thereby restoring the reading frame and generating a functional dystrophin protein [119,181,183]. Both gentamicin and negamycin have been examined in *mdx* mice, whereby low levels of dystrophin expression are observed. Gentamycin has been tested in DMD patients, where earlier clinical trials saw contradictory and inconclusive results and later trials saw only low levels of dystrophin expression in 50% of patients [117].

Subsequently, a high-throughput screen has identified PTC124, a small-molecule able to induce read-through of premature stop codons, which has shown significant promise in both *in vitro* and *in vivo* studies [119]. A major concern of this drug is the possibility of off-target effects [183], though there is no indication of this as of yet [119]. Nonetheless, positive preclinical results have led to PTC124, or ataluren, undergoing clinical trials, whereby the drug was well tolerated and most importantly, read-through of normal stop codons did not occur [120]. Further trials showed no significant functional outcome, though interestingly, several subgroups were positively affected by treatment. There was no mention of the heart [121,122] (clinicaltrials.gov, identifier: NCT01826487) (see Table 1). The results from this clinical trial clearly show that a better understanding regarding subgroups of patients and their functional outcomes is needed.

Other studies have aimed at identifying more effective drugs to induce read-through. A sensitive small-molecule screen was performed, which identified compounds RTC13 and RTC14. These compounds were subsequently administered intramuscularly and intravenously in *mdx* mice, and RTC13 demonstrated better efficacy (restoring dystrophin in skeletal muscle and especially the heart) than gentamicin, PTC124 and RTC14. In addition, muscle function was improved and serum CK levels were decreased in *mdx* mice treated with RTC13 [123]. RTC13 provides a promising new therapeutic molecule for patients with a premature stop codon.

### 4.3. Viral Gene Therapy

Another therapeutic strategy undergoing development is the use of viral vectors to deliver a dystrophin transgene to the cell. Given dystrophin’s enormous size, multiple studies have endeavored to distinguish the integral components of the dystrophin gene to develop a shortened truncated version that may be inserted into the viral capsid (mini- or microdystrophin) [184]. Several viruses have been considered as a potential delivery vector, including lentivirus, adenovirus and adeno-associated virus (AAV; see Table 1).

In order to safely apply the AAV virus, it has been modified to exclude all viral genes, but leave essential repeats for replication (recombinant AAV, rAAV) [182]. The AAV vector requires co-transfection of a vector carrying components necessary for replication. Different AAV serotypes exhibit different tropism [185], of which rAAV2/AAV8, rAAV6 and rAAV9 have shown particular promise in the *mdx* heart when using mini- or micro-dystrophin. rAAV6 microdystrophin was successful at transfection and induction of dystrophin in TA, diaphragm and heart in young *mdx* mice. Interestingly, however, heart function did not recover even though dystrophin was restored in the heart and heart mass was normal [129]. Injection of old *mdx* mice also produced positive results [130], suggesting that even though *mdx* muscles had been significantly damaged by the disease, treatment was still capable of improving some muscle pathology [130].

rAAV9 has proven to be particularly efficient in transfecting the heart [132]. Mini-dystrophin rAAV9 administered intravenously in golden retriever muscular dystrophy (GRMD) dogs revealed varied dystrophin expression (between 15% and 100% depending on the muscle analyzed) and was also present in the heart [131]. Micro-dystrophin rAAV9 administration in young and adult *mdx* mice also induced dystrophin expression and improved cardiac function [132,133]. Despite these positive results, immunological responses to the AAV virus have still been observed in larger animals and humans [180,186]. Moreover, high titers of virus are necessary to induce effective dystrophin expression in muscle cells. In an effort to reduce this, chimeric vectors such as rAAV2/8 vector containing a codon-optimized microdystrophin have been generated and intravenously injected in 10 week old *mdx* mice. Lower titer levels of the virus resulted in almost 100% transfection, effective dystrophin expression in both skeletal muscle and heart and improved muscle function. Moreover, no immunological response was observed [128].

Overall, viral vectors are promising candidates for the delivery of the dystrophin gene. However, special care should be taken regarding immunogenicity as the human population carries antibodies towards many prevalent viruses, such as AAV [182,187], though immunosuppression may be implemented to reduce this risk [188]. Moreover, incorrect insertions of the virus into the host genome may occur with vectors such as lentiviruses [182]. Lastly, repeat administration is problematic due to the presence of neutralizing antibodies from the first administration [180]. Further research is needed to eliminate these concerns.

### 4.4. Cell-Based Therapy

With cell-based therapy, healthy dystrophin-expressing cells are transplanted into DMD patient tissue which not only integrate into healthy adult muscle cells, but ideally also co-populate the satellite cells. These cells can be derived either from healthy precursor cells or from patient cells that are genetically modified *in vitro* [189]. Several different muscle precursor cells have been explored for therapeutic purposes in DMD, namely myoblasts, fibroblasts, bone-marrow derived stem cells, CD133+ stem cells and mesangioblasts (see Table 1) [134,135,136,137,138,139,140,141,190,191].

A more recent shift to the use of induced pluripotent stem (iPS) cells has been observed. Indeed, human iPS cells have been differentiated into muscle precursor cells and injected into TA muscles of immunocompromised *mdx* mouse models [143]. Following injection, dystrophin protein was restored, regeneration led to higher dystrophin levels and functional tests showed improvement [143,144]. These experiments illustrated that iPS cells were able to integrate with muscle cells and settle in the satellite cell population.

Several groups have aimed at utilizing genetically modified patient precursor cells as treatment. This would circumvent immunological suppression needed when introducing foreign material into the body. The genome of the cells are modified *in vitro* with the use of nucleases [192,193,194,195], whereby more recently, TALENs and CRISPR-cas9 have been used for splicing correction of the dystrophin gene. They were utilized in iPS cells *in vitro* to induce exon skipping, shift the reading frame or insert the missing exon with successful results. Subsequently, these iPS cells were differentiated into skeletal muscle cells and dystrophin expression was observed. The knock-in of the missing exon was most effective [193]. Given the novelty of the work, the study focused on skeletal cells and did not encompass the cardiomyocytes, but the results offer interesting prospects for dystrophin heart restoration.

Overall, utilizing genetically modified cells from patients would be a more valuable treatment when considering immunological responses. However, the ability to induce and sustain adequate dystrophin levels remains difficult. Currently, delivery of progenitor cells is most successful if done intramuscularly [182], yet this would only allow the treatment of one muscle at a time and muscles like the diaphragm and the heart would not be restored. Additionally, high titers of modified cells are needed to significantly alter the phenotype and functionality. Several obstacles are yet to overcome, but this technique appears promising and may prove to be very effective.

### 4.5. Antisense Oligonucleotides

Antisense oligonucleotides (AONs) are another viable approach for treating DMD patients, and are specifically utilized in the skipping of exons with mutations or deletions in the dystrophin gene in order to correct the aberrant reading frame [196]. AONs are short, single stranded DNA sequences which are complementary to a target pre-mRNA splice site, thereby sterically blocking splice enhancers and skipping the desired exon which produces a functional, albeit shorter, dystrophin protein [197]. This therapy is capable of treating ~83% of DMD patients with dystrophin mutations, specifically 79% of patients with deletions, 91% with small mutations and 73% with duplications [198]. A significant shortfall of this therapy is that AONs can only specifically target one exon at a time and therefore current efforts are focused on exons which would assist the greatest numbers of patients [198]. 

The predominant AON chemistries which are currently undergoing clinical evaluation are phosphorodiamidate morpholino oligomers (PMOs) [149] and 2′O methyl phosphorothioate (2′OMePS) [146] and both have shown promising results in the clinic (see Table 1) [146,149,153,199]. Although these chemistries demonstrate potential for treating DMD, the confounding issues are their relatively poor systemic delivery and their inability to restore dystrophin in the heart [146,149,200,201]. More recently, a new AON chemistry, tricyclo-DNA, has demonstrated marked preclinical success and was capable of restoring dystrophin in skeletal muscle and heart, with low levels in the brain of DMD mouse models. However, it should be noted that these positive findings required multiple dose administration and very high doses (200 mg/kg per week) [154].

In an effort to improve the delivery capacity of AONs and reduce the dose required, short peptide sequences, ‘cell penetrating peptides’ (CPPs), have been chemically conjugated to neutral AONs such as PMO to facilitate their delivery across the plasma membrane. A multitude of studies have investigated peptide conjugated AONs in the context of DMD and can broadly be classified into 3 distinct groups namely arginine rich, Pip (PNA/PMO internalization peptide), and phage and chimeric peptides.

Most importantly the arginine rich and Pip peptides have demonstrated cardiac dystrophin expression. Arginine rich CPPS conjugated to PMO were the first to show activity in the heart [163] and indeed further showed that sustained treatment improved cardiac function and resistance to dobutamine stress [158]. More recently, the Pip6 series has demonstrated dystrophin expression in the heart at very low doses (12.5 mg/kg) [166] and prevented exercise-induced cardiomyopathy following long-term treatment [167]. The phage peptides have been less successful in restoring cardiac dystrophin and function, with the exception of a 7-mer phage (conjugated to 2′OMePS), which resulted in exon skipping in multiple tissues including heart [172].

Another method to improve delivery of charged AONs is to encapsulate them in liposomes [202] or nanoparticles. More recent advancements have given rise to ZM4 nanoparticles bound to AONs which demonstrated widespread distribution to skeletal muscle and heart, thus highlighting the potential of this delivery method. Other new and innovative technologies being investigated include bispecific splice oligonucleotides targeting dystrophin and myostatin regulation [203], and knocking-in of deleted exons using meganucleases [195]. Given the current developments of AON therapy in the DMD field, it is no surprise that such a variety of novel approaches are being developed for the treatment of DMD.

To summarize, with the exception of tricyclo-DNA, naked AONs have demonstrated limited systemic delivery and an inability to restore dystrophin in the heart, whereas conjugation to CPPs or the use of encapsulation technologies have significantly improved upon this. From a CPP perspective, both poly-arginine and Pip CPPs have displayed widespread dystrophin restoration in multiple skeletal muscle tissues and the heart. Nanoparticles are another promising delivery strategy, particularly for charged AONs such as 2′OMePS. This field is rapidly evolving and it is hoped that one or more of these technologies is advanced to clinical trial in the near future.

Whilst these experimental therapies have inspired new hope for patients and families affected by DMD, the feasibility of clinically translating some of these therapies has been brought into question. Not only should species differences be considered in terms of cellular processes and pathways, but also concerning immunological responses. Mouse models of DMD are somewhat resilient, and studies in dog models have highlighted inevitable obstacles particularly due to our complex immune system. This is impossible to avoid given the requirement of a systemic approach to treat a disease which is devastating to the entire body. Not only is preventing an immunological response particularly relevant for cell-based therapies and viral vectors, but also for peptide-conjugated AONs which may cause complications. Certainly, long term administration of ‘naked’ PMO did not elicit an immunological response. Other therapies such as aminoglycosides may induce off-target or other toxicological affects. Ultimately, in order to substantially ameliorate or abrogate this disease, targeting the underlying defect, specifically the lack of dystrophin protein, is required. Current pharmacological interventions can only delay or partially ameliorate the symptoms of this disease. Therefore it is imperative that one or more, or indeed a new therapy altogether, is able to overcome these obstacles and by-pass our innate immune system.

## 5. Conclusions

Our current understanding of Duchenne muscular dystrophy has increased significantly over the last few decades. The absence of the dystrophin protein gives rise to a complex pathology with the involvement of many different processes in the cell all playing an intricate part in further disease progression. Much research has already been performed which indicate the importance of Ca^2+^, NO, mitochondria and other second messengers and organelles in the cell. However, given the gaps that still remain in our knowledge, it seems likely that other unknown proteins or pathways also affect disease pathology. Furthermore, as dystrophin is expressed body-wide, it is important that individual systems such as the heart are considered, as specific mechanistic findings from skeletal muscle pathology may not necessarily be applied to the heart. Moreover, the comparison of molecular aspects of dystrophin function between different cell types could provide valuable information.

The knowledge gained from improved understanding of molecular pathological pathways provides more ways of developing and assessing the effectiveness of treatment. With the availability of steroids and other drugs, improvements have been made to ameliorate the skeletal muscle phenotype. Whilst pharmacological treatments to stabilize cardiac function, such as ACE inhibitors and beta-blockers, are in use, mortality from cardiorespiratory complications in DMD patients remains high. Moreover, whilst some experimental therapies are under development, these may not benefit cardiac aspects of the disease and most clinical trials to date have focused exclusively on the beneficial effects of drug treatment on skeletal muscle function, without considering the impact on heart function. Therefore, an increased focus on the heart is essential, both in terms of existing treatments and the effects on heart function, but also more critically on novel treatments that could be developed to specifically restore dystrophin or upregulate utrophin in cardiac muscle, resulting in clinical benefit for patients.
